# Effectiveness of PD-1 inhibitor-based first-line therapy in Chinese patients with metastatic gastric cancer: a retrospective real-world study

**DOI:** 10.3389/fimmu.2024.1370860

**Published:** 2024-06-12

**Authors:** Yichun Duan, Jielang Li, Shuang Zhou, Feng Bi

**Affiliations:** Division of Abdominal Cancer, Department of Medical Oncology, Cancer Center and Laboratory of Molecular Targeted Therapy in Oncology, West China Hospital, Sichuan University, Chengdu, Sichuan, China

**Keywords:** PD-1 inhibitors, gastric cancer, immunotherapy, real-word study, drug response biomarkers

## Abstract

**Objective:**

Programmed cell death protein-1 (PD-1) inhibitor-based therapy has demonstrated promising results in metastatic gastric cancer (MGC). However, the previous researches are mostly clinical trials and have reached various conclusions. Our objective is to investigate the efficacy of PD-1 inhibitor-based treatment as first-line therapy for MGC, utilizing real-world data from China, and further analyze predictive biomarkers for efficacy.

**Methods:**

This retrospective study comprised 105 patients diagnosed with MGC who underwent various PD-1 inhibitor-based treatments as first-line therapy at West China Hospital of Sichuan University from January 2018 to December 2022. Patient characteristics, treatment regimens, and tumor responses were extracted. We also conducted univariate and multivariate analyses to assess the relationship between clinical features and treatment outcomes. Additionally, we evaluated the predictive efficacy of several commonly used biomarkers for PD-1 inhibitor treatments.

**Results:**

Overall, after 28.0 months of follow-up among the 105 patients included in our study, the objective response rate (ORR) was 30.5%, and the disease control rate (DCR) was 89.5% post-treatment, with two individuals (1.9%) achieving complete response (CR). The median progression-free survival (mPFS) was 9.0 months, and the median overall survival (mOS) was 22.0 months. According to both univariate and multivariate analyses, favorable OS was associated with patients having Eastern Cooperative Oncology Group performance status (ECOG PS) of 0–1. Additionally, normal baseline levels of carcinoembryonic antigen (CEA), as well as the combination of PD-1 inhibitors with chemotherapy and trastuzumab in patients with human epidermal growth factor receptor 2 (HER2)-positive MGC, independently predicted longer PFS and OS. However, microsatellite instability/mismatch repair (MSI/MMR) status and Epstein-Barr virus (EBV) infection status were not significantly correlated with PFS or OS extension.

**Conclusion:**

As the first-line treatment, PD-1 inhibitors, either as monotherapy or in combination therapy, are promising to prolong survival for patients with metastatic gastric cancer. Additionally, baseline level of CEA is a potential predictive biomarker for identifying patients mostly responsive to PD-1 inhibitors.

## Introduction

Gastric cancer ranks among the most prevalent cancers globally, standing as the fourth most common cause of cancer-related deaths in 2020, with an estimated 769,000 fatalities (1). The incidence of gastric cancer exhibits regional variation, with a higher prevalence observed in East Asia, particularly in China. In 2022, it ranked as the fifth most common cancer in China, with estimated incidence and mortality rates of 10.5% and 12.4%, respectively (2). Regrettably, alongside the high incidence of this disease, the majority of patients are diagnosed at an advanced stage upon detection. This is often due to the early clinical symptoms of gastric cancer being mild and easily overlooked. In patients with advanced or metastatic gastric cancer (AGC/MGC), conventional systemic chemotherapy remains the predominant treatment option. Commonly used regimens typically revolve around fluorouracil-based and platinum-based treatments such as SOX (oxaliplatin + S-1), XELOX (oxaliplatin + capecitabine), FOLFOX (calcium folinate + fluorouracil + oxaliplatin) and so on. However, chemotherapy shows limited effect, with a median survival of 11–12 months (3). For human epidermal growth factor receptor 2 (HER2)-positive patients, the recommended first-line treatment option for advanced cancers is the combination of the anti-HER2 antibody trastuzumab with chemotherapy (4, 5). Nonetheless, patient benefit remains constrained, underscoring the imperative to investigate further treatment alternatives.

Immune checkpoint inhibitors (ICIs), including antibodies against programmed cell death protein-1 (PD-1) or its ligand (PD-L1), have been rapidly developed over the past few years and are now established treatments for chemotherapy-refractory gastric cancers (cancers that progress after two or more lines of chemotherapy) (6, 7). Previous clinical studies have confirmed that some gastric cancer patients may benefit from PD-1 inhibitor therapy. Regarding first-line treatments for AGC/MGC, the KEYNOTE-062 trial, the first global randomized phase III trial comparing the efficacy and safety of pembrolizumab, a humanized anti-PD-1 monoclonal antibody, with chemotherapeutic agents in AGC/MGC, demonstrated that pembrolizumab alone was not inferior to chemotherapy [median overall survival (mOS): 10.6 vs. 11.1 months, hazard ratio (HR): 0.91, 95% confidence interval (CI): 0.69–1.18]. Moreover, pembrolizumab even extended OS in patients with a combined positive score (CPS) of 10 or greater (mOS: 17.4 vs. 10.8 months, HR: 0.69, 95% CI: 0.49–0.97). However, pembrolizumab in combination with chemotherapy did not result in an improvement in OS or progression-free survival (PFS) compared to chemotherapy, either in patients with CPS ≥1 or CPS ≥10 (8). In another phase III trial, KEYNOTE-859, pembrolizumab in combination with chemotherapy prolonged patients’ OS and PFS compared to chemotherapy alone (9). For nivolumab, another clinically utilized PD-1 inhibitor, the combination of nivolumab with a chemotherapeutic agent notably enhanced PFS across all CPS subgroups in both the CheckMate 649 and ATTRACTION-4 trials. Additionally, in the CheckMate 649 trial, overall survival was prolonged. However, there was no significant difference between the two treatment modalities in the ATTRACTION-4 trial (10, 11). Regarding second- and third-line therapy, two trials conducted globally and in Asia showed favorable activity and a manageable safety profile for PD-1 inhibitors in patients with refractory advanced gastric cancer or gastroesophageal junction cancer (GEJC) who had received at least two prior therapies (12, 13). In contrast, another phase III trial, JAVELIN Gastric 300, which explored the efficacy of avelumab, a complete human IgG1 monoclonal antibody, as maintenance therapy in patients with AGC/GEJC, led to the conclusion that avelumab did not improve OS or PFS compared to chemotherapy as third-line treatment (14).

Most of the above trials have demonstrated the effectiveness of PD-1 inhibitors in AGC/MGC, but the treatment response varies among the studies [objective response rate (ORR): 20%-65.1%] (8–10, 15). Findings on the benefits of OS, PFS, and ORR were also inconsistent. The clinical application of PD-1 inhibitor-based therapies is increasingly prevalent; however, there is limited real-world evidence regarding the use of PD-1 inhibitors as monotherapy or in combination treatments. This study endeavors to investigate the effectiveness of PD-1 inhibitor monotherapy or combination therapy in patients with metastatic gastric cancer through analysis of real-world clinical data. Furthermore, we conducted an additional analysis to explore the relationship between clinical characteristics and treatment efficacy, aiming to identify potential biomarkers for predicting the effectiveness of anti-PD-1 therapy.

## Materials and methods

### Study population

This retrospective, single-center study involved patients treated with PD-1 inhibitors monotherapy or a combination of chemotherapeutic agents and targeted agents, as documented by their medical history. We retrospectively collected information on MGC patients who received different PD-1 inhibitor-based treatments between January 2018 and December 2022 at West China Hospital of Sichuan University. The inclusion criteria were as follows: 1) pathologically confirmed gastric cancer; 2) no surgical options available after the initial diagnosis, or disease progression, metastasis, or recurrence after surgical cure; 3) receiving PD-1 inhibitor monotherapy or combination therapy in first line; 4) receiving at least 2 cycles of PD-1 inhibitor-based therapy; and 5) at least one measurable lesion; The major exclusion criteria included: 1) involvement of primary malignant tumors from other systems; and 2) loss to follow up for unknown reasons. We retrieved and integrated clinical information and basic characteristics from the records of the enrolled patients, including age, sex, HER2 expression status, Epstein-Barr virus (EBV) infection status, microsatellite instability/mismatch repair (MSI/MMR) status, carcinoembryonic antigen (CEA) baseline level, Eastern Cooperative Oncology Group performance status (ECOG PS), tumor stage, pathology type, metastatic organs, previous immunotherapy status, and treatment regimen. Patients were included regardless of their statuses of MMR proteins through immunohistochemical method or MSI through Next-Generation sequencing testing. The study was approved by the Ethics Committee of West China Hospital of Sichuan University, in accordance with the Declaration of Helsinki and the International Ethical Guidelines for Human Biomedical Research (Ethics Approval Number: 2023–2073).

### Study design and treatment regimens

The chemotherapy regimens used SOX (oxaliplatin + S-1), TP (albumin paclitaxel + cisplatin), and XELOX (oxaliplatin + capecitabine), and the targeted drugs was the injectable trastuzumab (herceptin) for HER2-positive patients. The regimen of each PD-1 inhibitor for the concurrent use with chemotherapy drugs or targeted drugs is as follows: pembrolizumab (200mg), cedilimumab (200mg), tislelizumab (200mg) or toripalimab (240mg) for IV infusion every 3 weeks; or carrelizumab (200mg) or nivolumab (3mg/kg) for IV infusion every 2 weeks.

### Assessment

The primary endpoint of this study was overall survival, defined as the time from the start of the patient’s PD-1 inhibitor-based therapy to the time of death from any cause, and the secondary endpoint was progression-free survival, defined as the time from the start of the patient’s PD-1 inhibitor-based therapy to the time of disease progression or death from any cause. Based on the Response Evaluation Criteria in Solid Tumors 1.1, the additional efficacy assessment metrics included: complete response (CR), partial response (PR), stable disease (SD), progressive disease (PD), the objective response rate which is the sum of the proportions of complete response and partial response, as well as the disease control rate (DCR) which is defined as the proportion of cases achieving remission (CR+PR) and disease stability (SD) after treatment, representing the percentage of patients without disease progression.

### Statistical analysis

Variables are presented as median (range) for continuous variables and numbers (%) for categorical variables. Relationships between categorical variables were examined by means of the Chi-square test. Univariate and multivariate analyses were performed to evaluate the prognostic impact on PFS and OS. The Kaplan–Meier method was applied to estimate survival probabilities and the log-rank test was carried out to assess heterogeneity within each prognostic factor. The HR was estimated using the Cox proportional hazard model. Cox proportional hazards regression analysis was carried out as a multivariate analysis to assess the prognostic value of the markers adjusted for the possible confounding effect of all the other factors included in the same model. All the statistical tests were two-sided, and differences for which p values were less than 0.05 were considered significant. All statistical analyses were performed using SPSS 27.0 software.

## Results

### Patients’ characteristics and treatment

Our study included 105 patients whose median age was 55 years (range: 27–85). Among these patients, 51 (48.6%) were male, 103 (98.1%) had an ECOG PS score of 0–1, 97 (92.4%) had adenocarcinomas, 72 (68.6%) had poorly differentiated cancers. The numbers of tumors that metastasized to the liver, lung, lymph nodes, bone and peritoneum/pleura were 28 (26.7%), 9 (8.6%), 80 (75.5%), 8 (7.6%) and 48(11.9%), respectively. The PD-1 inhibitors used by patients included nivolumab (n=46, 43.8%), pembrolizumab (n=4, 3.8%), cedilimumab (n=26, 24.8%), carrelizumab (n=20, 19.0%), tislelizumab (n=7, 6.7%), toripalimab (n=2, 1.9%). A total of 3 (2.9%) individuals received treatment with PD-1 inhibitors alone, 92 (87.6%) received combined PD-1 inhibitor and chemotherapy, and 10 (9.5%) received combined PD-1 inhibitor, chemotherapy and targeted therapy. A total of 10 (9.5%) had positive HER2 status, 5 (4.8%) had deficient mismatch repair/microsatellite instability-high (dMMR/MSI-H) status, and 6 (5.7%) had positive EBV status, and 39 (37.1%) had CEA baseline level greater than normal baseline level. **Table 1** shows more detailed information.

**Table 1:** Patients and tumor characteristics.

Characteristic	N (%)
Age (range)^*^	55 (27–85)
≤60y	65 (61.9)
>60y	40 (38.1)
Gender	
Male	51 (48.6)
Female	54 (51.4)
ECOG PS	
0–1	103 (98.1)
≥2	2 (1.9)
Pathological type	
Adenocarcinoma	97 (92.4)
Non-adenocarcinoma	8 (7.6)
Histological differentiation	
Poorly	72 (68.6)
Moderately/Well	33 (31.4)
HER2 status	
Negative	84 (80.0)
Positive	10 (9.5)
Unknown	11 (10.5)
MSI/MMR status	
pMMR/MSS	75 (71.4)
dMMR/MSI-H	5 (4.8)
Unknown	25 (23.8)
EBV status	
Negative	72 (68.6)
Positive	6 (5.7)
Unknown	27 (25.7)
CEA level	
≤ normal baseline CEA levels	66 (62.9)
> normal baseline CEA levels	39 (37.1)
Metastatic site	
Liver	28 (26.7)
Lung	9 (8.6)
Lymph node	80 (75.5)
Bone	8 (7.6)
Peritoneum/Pleura	48 (11.9)
Anti-PD-1 therapy	
Monotherapy	3 (2.9)
Plus chemo	92 (87.6)
Plus chemo + trastuzumab	10 (9.5)
Anti-PD-1 therapy type	
Nivolumab	46 (43.8)
Pembrolizumab	4 (3.8)
Cedilimumab	26 (24.8)
Carrelizumab	20 (19.0)
Tislelizumab	7 (6.7)
Toripalimab	2 (1.9)

* The difference between the maximum and minimum values.

ECOG PS Eastern Cooperative Oncology Group performance status, HER2 human epidermal growth factor receptor 2, MSI/MMR microsatellite instability/mismatch repair, pMMR/MSS proficient mismatch repair/microsatellite stable, dMMR/MSI-H deficient mismatch repair/microsatellite instability-high, EBV Epstein-Barr virus, CEA carcinoembryonic antigen, PD-1 programmed cell death protein-1, chemo chemotherapy.

### Efficacy

**Figure 1:**
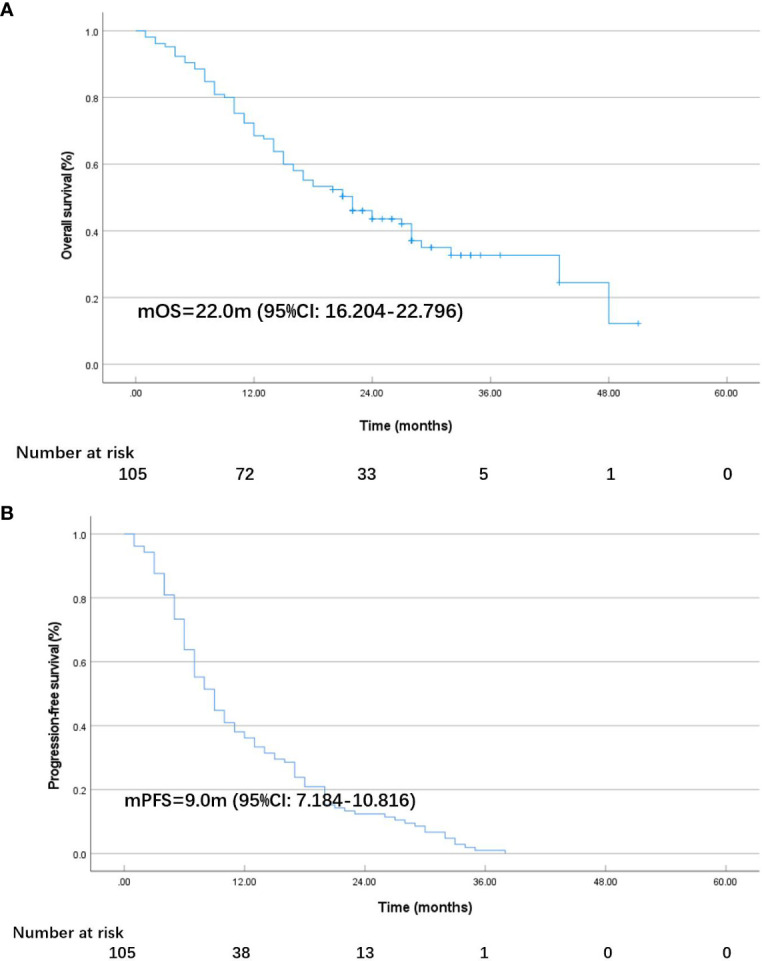
K-M plot of overall survival (OS) **(A)** and progression-free survival (PFS) **(B)** in all patients.

As of October 17, 2023, among the 105 individuals enrolled in the study, 2 (1.9%) patients achieved CR, and an ORR of 30.5% and a DCR of 89.5% were observed in the total population. Specific information can be found in **Table 2**. After 28.0 months (95% CI: 25.114–30.886) of follow-up, the median OS in the total population was 22.0 months (95% CI: 16.204–22.796; **Figure 1A**), and the median PFS was 9.0 months (95% CI: 7.184–10.816; **Figure 1B**). At the cut-off date, 40 patients were still alive.

**Table 2:** Response outcome.

Response	N(%)
CR	2 (1.9)
PR	30 (28.6)
SD	62 (59.0)
PD	11 (10.5)
ORR(CR+PR)	32 (30.5)
DCR(CR+PR+SD)	94 (89.5)

CR complete response, PR partial response, SD stable disease, PD progressive disease, ORR objective response rate, DCR disease control rate.

### Univariate analysis and multivariate analysis

**Figure 2:**
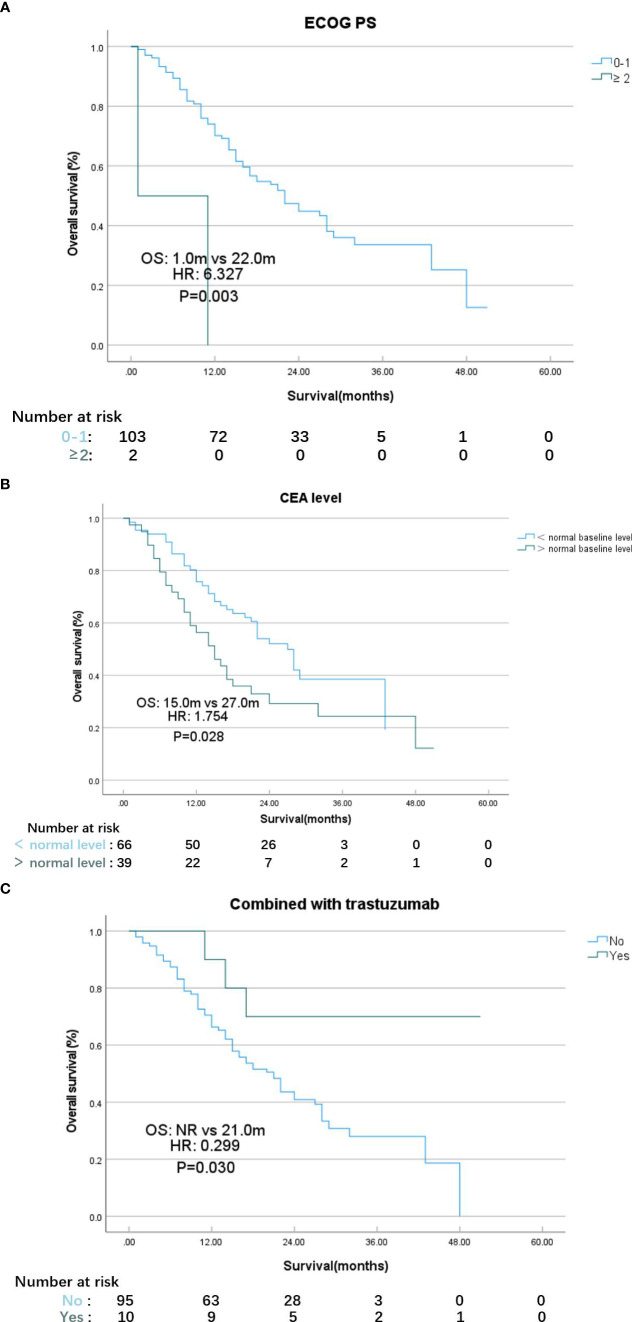
K-M plot of overall survival for patients with different ECOG PS scores **(A)**, CEA level **(B)**, therapy strategy **(C)**. **(A)** *ECOG PS* Eastern Cooperative Oncology Group performance status. **(B)** *CEA* carcinoembryonic antigen. **(C)** *NR* not reached.

According to the results of the univariate analysis and multivariate analysis, favorable prognostic factors for OS included an ECOG PS score <2, CEA at the normal level, and the addition of targeted therapy for HER2-positive MGC patients (**Tables 3**, **4**). The median OS for patients with ECOG PS score of 0–1 was 22.0 months, while for those with a score ≥2, it was 1.0 months (HR: 6.327, 95% CI: 1.502–26.648; p=0.012, **Figure 2A**). In the subgroups, patients with elevated CEA levels at baseline exhibited poorer immunotherapy responsiveness than those with normal CEA level: mOS (15.0 vs. 27.0 months, HR: 1.754, 95% CI: 1.031–2.985; p=0.038, **Figure 2B**) and mPFS (6.0 vs. 9.0 months, HR: 1.533, 95% CI: 0.992–2.369; p=0.054). Additionally, the combination of PD-1 inhibitors with chemotherapy and trastuzumab, compared to PD-1 inhibitors monotherapy or combined with chemotherapy alone, may result in a better mOS (>28.0 vs. 21.0 months, HR: 0.299, 95% CI: 0.093–0.962; p=0.043, **Figure 2C**, **Table 3**) and mPFS (12.0 vs. 8.0 months, HR: 0.597, 95% CI: 0.308–1.158; p=0.127, **Table 4**), however, PFS were not significantly different between the two subgroups.

**Table 3:** Univariate analysis and multivariate analysis of clinical variables for the prediction of overall survival.

	Univariate Cox model			Multivariate Cox model		
Characteristics	HR	95%CI	P value	HR	95%CI	P value
**Gender**	1.108	0.678–1.810	0.682			
Male vs Female						
**Age**	0.940	0.573–1.544	0.808			
>60 vs ≤60						
**ECOG PS**	6.327	1.502–26.648	**0.012**	5.439	1.153–25.645	**0.032**
≥2 vs 0–1						
**Pathological type**	0.431	0.135–1.378	0.156			
Non-adenocarcinoma vs Adenocarcinoma						
**Histological differentiation**	0.711	0.407–1.241	0.230			
Moderately/Well vs Poorly						
**HER2 status**	0.310	0.096–1.0	0.050			
Positive vs Negative						
**MSI/MMR status**	0.546	0.132–2.254	0.403			
Positive vs Negative						
**EBV status**	0.193	0.026–1.405	0.104			
Positive vs Negative						
**CEA level > normal baseline CEA levels**	1.754	1.031–2.985	**0.038**	1.926	1.077–3.444	**0.027**
Yes vs No						
**Liver metastasis**	1.040	0.601–1.80	0.890			
Yes vs No						
**Lung metastasis**	1.0	0.425–2.353	1.0			
Yes vs No						
**Bone metastasis**	1.330	0.533–3.317	0.541			
Yes vs No						
**Lymph node metastasis**	0.870	0.504–1.50	0.616			
Yes vs No						
**Peritoneum/Pleura metastasis**	1.512	0.922–2.481	0.101			
Yes vs No						
**Anti-PD-1+ trastuzumab**	0.299	0.093–0.962	**0.043**	0.283	0.084–0.947	**0.041**
Yes vs No						

ECOG PS Eastern Cooperative Oncology Group performance status, HER2 human epidermal growth factor receptor 2, MSI/MMR microsatellite instability/mismatch repair, EBV Epstein-Barr virus, CEA carcinoembryonic antigen, PD-1 programmed cell death protein-1. bold value: this value < 0.05.

**Table 4:** Univariate analysis and multivariate analysis of clinical variables for the prediction of progression free survival.

	Univariate Cox model			Multivariate Cox model		
Characteristics	HR	95%CI	P value	HR	95%CI	P value
**Gender**	0.928	0.628–1.369	0.705			
Male vs Female						
**Age**	0.720	0.482–1.077	0.110			
>60 vs ≤60						
**ECOG PS**	2.557	0.623–10.492	0.192			
≥2 vs 0–1						
**Pathological type**	0.677	0.328–1.397	0.291			
Non-adenocarcinoma vs Adenocarcinoma						
**Histological differentiation**	0.874	0.569–1.342	0.538			
Moderately/Well vs Poorly						
**HER2 status**	0.628	0.322–1.224	0.172			
Positive vs Negative						
**MSI/MMR status**	1.076	0.428–2.702	0.877			
Positive vs Negative						
**EBV status**	0.632	0.274–1.460	0.283			
Positive vs Negative						
**CEA level > normal baseline CEA level**	1.533	0.992–2.369	0.054	12.423	1.377–4.266	**0.002**
Yes vs No						
**Liver metastasis**	0.998	0.637–1.563	0.993			
Yes vs No						
**Lung metastasis**	0.983	0.470–2.054	0.964			
Yes vs No						
**Bone metastasis**	1.327	0.642–2.742	0.445			
Yes vs No						
**Lymph node metastasis**	0.903	0.569–1.433	0.664			
Yes vs No						
**Peritoneum/Pleura metastasis**	1.713	1.146–2.561	**0.009**	1.295	0.787–2.131	0.309
Yes vs No						
**Anti-PD-1+ trastuzumab**	0.597	0.308–1.158	0.127	0.393	0.172–0.898	**0.027**
Yes vs No						

ECOG PS Eastern Cooperative Oncology Group performance status, HER2 human epidermal growth factor receptor 2, MSI/MMR microsatellite instability/mismatch repair, EBV Epstein-Barr virus, CEA carcinoembryonic antigen, PD-1 programmed cell death protein-1. bold value: this value < 0.05.

In the univariate analysis, considering that true effect of these factors may be masked by the effects of other confounding factors in single-factor analysis, we selected several indicators that were meaningful in the univariate analysis of OS for multivariate analysis, with favorable prognostic factors including CEA at the normal level (HR: 12.423, 95% CI: 1.377–4.266; p=0.002) and the addition of targeted therapy, trastuzumab, to immunotherapy (HR: 0.393, 95% CI: 0.172–0.898; p=0.027).

### Molecular features associated with OS and PFS

MSI/MMR status and EBV infection status are commonly used molecular biomarkers for immunotherapy. However, this study found no significant correlation between different MSI/MMR status (positive vs. negative) and EBV infection status (positive vs. negative) with changes in mOS (MSI/MMR: 24.0 vs. 22.0 months, p=0.403; EBV: >28.0 vs. 22.0 months, p=0.104) and mPFS (MSI/MMR: 6.0 vs. 9.0 months, p=0.877; EBV: 17.0 vs. 9.0 months, p=0.283). Therefore, these two biomarkers cannot currently be deemed predictive of responsiveness to immunotherapy.

## Discussion

Progressive gastric cancer has a poor prognosis and current treatment options are limited. With the rise of immunotherapy in recent years, PD-1 inhibitors such as nivolumab and pembrolizumab in combination with chemotherapy also have been recommended for treatment of metastatic gastric cancer (7, 16, 17). However, most previous conclusions were drawn from clinical trials, and there is considerable variation in patient outcomes, with ORR ranging from 20% to 65.1% (9, 10, 15, 18). Hence, it is imperative to investigate the real-world benefits that gastric cancer patients derive from first-line treatment with immune checkpoint inhibitors. To the best of our knowledge, this study represents a largest single-center real-world analysis among Chinese patients with MGC undergoing first-line treatment with anti-PD-1 therapy. Our findings suggest that first-line treatment with PD-1 inhibitors yields significant efficacy, particularly show that the median OS was 22.0 months (95% CI: 16.204–22.796), and the median PFS was 9.0 months (95% CI: 7.184–10.816) with an ORR of 30.5% and a DCR of 89.5%. Furthermore, compared to chemotherapy regimens alone in previous studies (OS:6.2–14 months, PFS: 4.3–12.1 months), the efficacy of chemotherapy combined with PD-1 inhibitors was indeed superior (3). Our patients obtained a longer OS.

It appears that patients may potentially benefit more from combination therapy with chemotherapy plus trastuzumab compared to PD-1 monotherapy or combination with chemotherapy alone. After a follow-up period of 28.0 months in our study, patients treated with PD-1 inhibitors exhibit a mOS of 21 months, while those receiving the combination of PD-1 inhibitors and trastuzumab remain alive, and thus have not yet reached their mOS. For HER2-positive patients, the current recommended standard treatment entails combining chemotherapy with trastuzumab. Recent studies have shown that adding PD-1 inhibitors to the combination of chemotherapy and trastuzumab can enhance efficacy in HER2-positive patients (19–21). Our study further supports this evidence. Nevertheless, due to the limited number of HER2-positive cases, further analysis and expansion of this patient subgroup are warranted.

Although anti-PD-1 treatment has improved patient prognosis compared to early studies, a considerable portion of patients still do not benefit from it, and there is also an increase of the risk of severe immune-related adverse effects. Therefore, researchers continue to explore and identify biomarkers to select the most suitable candidates for immunotherapy. Previous experiments have explored various biomarkers for predicting the efficacy of immunotherapy, including two gastric cancer genomic subtypes identified by The Cancer Genome Atlas (TCGA) studies: EBV infection status and MSI status (22). EBV infection can upregulate PD-L1 expression (23, 24), while DNA replication defects caused by dMMR/MSI-H result in the accumulation of mutations and the expression of new antigens, which may serve as potential targets for immune cells (25). Clinical trial findings suggest that patients with EBV infection or dMMR/MSI-H tumors may be more sensitive to immunotherapy (26–29). However, some experiments also demonstrate that these two biomarkers may not predict the response to immunotherapy in gastric cancer effectively (30–32). In this study, the ORR for two biomarkers EBV-positive and dMMR/MSI-H were 66.7% and 40%, respectively, which align with previous research findings (28, 29, 33). However, there were no significant statistical differences in OS and PFS between the subgroups. This lack of significance may be attributed to the low proportion of patients testing positive for these indicators. Consequently, the small sample size of these patients may have limited statistical power, underscoring the necessity to expand the sample size.

Currently, these biomarkers have limited clinical utility due to their uncertain predictive value, low positivity rates, and high cost. Therefore, we are striving to identify a more effective and convenient biomarker to predict the efficacy of immunotherapy. CEA is a widely used tumor marker in gastric cancer, playing a significant role in disease diagnosis and prognosis (34, 35). Additionally, research has demonstrated ability of CEA to predict the efficacy of PD-1 inhibitors, in non-small cell lung cancer. CEA values have been identified as predictors of patient responsiveness to immune checkpoint inhibitors (36, 37). In the current study, patients with CEA levels above the normal level had a mOS of 15.0 months, while those with normal CEA levels had a mOS of 27.0 months. Similarly, the mPFS was 6.0 months for patients with CEA levels above the standard level and 9.0 months for those with normal level, with statistically significant differences observed. It can be inferred that patients with CEA baseline levels below the standard threshold may derive greater benefits from immunotherapy. In the future, CEA levels might be utilized to predict patients’ response to anti-PD-1 therapy.

This study also has some limitations. Firstly, our study is a retrospective analysis, which inevitably introduces biases. Secondly, despite being the largest single-center, retrospective study to date, the sample size remains insufficient, for instance, efficacy comparisons among various PD-1 inhibitors were not feasible. Thirdly, due to the retrospective nature of the study, certain molecular markers in patients were not assessed, such as PD-L1 expression and tumor mutation burden (TMB), thus preventing analysis of these markers’ predictive value for treatment efficacy.

## Conclusion

In conclusion, our study illustrates the effectiveness of PD-1 inhibitor-based therapy for patients with metastatic gastric cancer. Additionally, we found that carcinoembryonic antigen shows promise as a predictor of immunotherapy efficacy.

## Data availability statement

The raw data supporting the conclusions of this article will be made available by the authors, without undue reservation.

## Ethics statement

The studies involving humans were approved by Biomedical Research Ethics Committee of West China Hospital of Sichuan University. The studies were conducted in accordance with the local legislation and institutional requirements. The ethics committee/institutional review board waived the requirement of written informed consent for participation from the participants or the participants’ legal guardians/next of kin because studies utilizing medical records were obtained from previous clinical consultations.

## Author contributions

YD: Writing – original draft, Writing – review & editing. JL: Writing – review & editing. SZ: Writing – review & editing. FB: Writing – review & editing.
